# The Early Diagnosis of Peritonitis

**Published:** 1904-05-28

**Authors:** 


### THE EARLY DIAGNOSIS OF PERITONITIS.

In a paper read before the New York Post-graduate Clinical Society, Dr. Forbes Hawkes [1] drew attention to the extreme importance of recognising the presence of peritonitis in its earliest stages. He holds that if the general practitioner would hand over his mild cases of peritonitis, from whatever cause, at an early period to the surgeon, more of those cases could be returned to his care afterwards, and that the patients so returning would be in a much better local and general condition after their operations than is the case at present. He points out that if the diagnosis of peritonitis be made only in the presence of the classical symptoms of abdominal pain and tenderness, constipation, vomiting, elevation of temperature, acceleration of pulse and abdominal distension, the larger proportion of mild cases of peritonitis will escape detection. There is one sign, however, that is of the greatest value in this connection, and that is abdominal rigidity. With few exceptions, rigidity of the abdominal muscles signifies peritonitis, mild forms of peritoneal inflammation being manifested by the lightest degree of localised abdominal rigidity. In order to detect the slightest grades it is essential to bestow much care on the method of examining. The patient must be lying on his back, stretched out perfectly straight, with the knees bent up equally and in a comfortable degree of flexion, preferably quite close together; the hands must be at the sides in exactly the same position. The head must be kept absolutely in the middle line, the mouth open and the breathing regular and moderate. Preferably the head and chest are slightly flexed. The room and the examiner's hand must be comfortably warm. The examination is carried out as follows: with the tips of the fingers fairly close together, the terminal palmar portions of them are made to sink down through the fatty layer overlying the muscles, rather suddenly but quietly, until the muscular layer is distinctly felt. The amount of rigidity present can best be stated as being the amount of resistance which the muscle offers to the fingers, which are both trying to depress the muscle and to sink into its substance. The examination is made systematically with the two hands. First the lower portions of the recti are examined, then the middle, and then the upper portions, and any difference between the two sides, or between different segments on the same side are carefully noted. In the same way the rest of the abdominal wall is palpated.

Not only is rigidity of value as an early sign of peritonitis, but it is an extremely useful aid to localising the inflammatory lesion, so that the surgeon may be guided to the right place for his incision.

Conditions other than peritonitis which may lead to rigidity are the various colics, simple, lead, calculous (biliary, renal and ureteral), and appendicular; the lightning pains of locomotor ataxia; pneumonia and diaphragmatic pleurisy.

[1] Post Graduate, April.

